# The efficacy of sodium glucose co-transport-2 inhibitors on glycemic control for patients with type 1 diabetes mellitus: A protocol for systematic review and meta-analysis

**DOI:** 10.1097/MD.0000000000026417

**Published:** 2021-07-09

**Authors:** Yajie Zhang, Ping Gan, Yanan Huo

**Affiliations:** Department of Endocrinology, Jiangxi Provincial People's Hospital Affiliated to Nanchang University, Nanchang, Jiangxi, PR China.

**Keywords:** meta-analysis, protocol, SGLT2 inhibitors, type 1 diabetes mellitus

## Abstract

**Background:**

Currently, there are a number of sodium glucose co-transport-2 (SGLT2) inhibitors that are under development or in clinical trials. Prior meta-analyses had established the safety and efficacy of SGLT2 inhibitors in type 1 diabetes mellitus (T1DM), but with low level of evidences and inconsistent conclusions. However, recently many new randomized clinical trials (RCTs) have been published, we hence try to design a study protocol to assess the effect of SGLT2 inhibitors on cardiovascular events via a comprehensive meta-analysis of data from much more RCTs, including sensitivity and subgroup analyses.

**Methods:**

We will follow the Preferred Reporting Items for Systematic Reviews and Meta-Analyses (PRISMA) reporting guidelines to conduct this meta-analysis. Two investigators will perform a systematic search of scientific literature in the databases (from conception through June 12, 2021), including PubMed, Embase, and Cochrane Central Register of Controlled Trials. This meta-analysis will be conducted using RevMan statistical software. The risk of bias for each included study will be assessed using the Cochrane Risk of Bias Assessment Tool.

**Results:**

Our protocol is conceived to test the hypothesis that SGLT2 inhibitors could lead to better outcomes in patients presenting with T1DM.

**Registration number:**

10.17605/OSF.IO/ZD8WX.

## 1 Introduction

Diabetes is the seventh leading cause of death worldwide, and its prevalence and incidence are increasing. Type 1 diabetes mellitus (T1DM) presents many challenges for patients and prescribers when trying to control blood glucose levels. The lack of novel treatment options, coupled with the challenges of long-term insulin therapy, makes it difficult for patients with T1DM to reach and maintain hemoglobin A1c goals, which increases the risk of complications.^[1,2]^ In addition, tolerance to insulin therapy depends on the relationship between the adverse effects of balancing weight gain and hypoglycemia and the achievement of blood glucose goals.^[3]^

Among the drugs currently being tested as adjuncts to insulin therapy in randomized clinical trials (RCTs), sodium glucose co-transport-2 (SGLT2) inhibitors appear promising because they are unique and independent of the mechanism of action of insulin.^[4]^ Overall, SGLT2 inhibitors significantly improve major cardiometabolic parameters (hemoglobin A1c, fasting glucose, blood pressure, lipid distribution, body weight) and are expected to be successful in cardiovascular disease and diabetic kidney disease. Therefore, in addition to insulin, they may be one of the most attractive treatment options for people with T1DM.^[5,6]^

Currently, there are a number of SGLT2 inhibitors that are under development or in clinical trials.^[6–8]^ Prior meta-analyses had established the safety and efficacy of SGLT2 inhibitors in T1DM, but with low level of evidences and inconsistent conclusions.^[9,10]^ However, recently, many new RCTs has been published, we hence try to design a study protocol to assess the effect of SGLT2 inhibitors on cardiovascular events via a comprehensive meta-analysis of data from much more RCTs, including sensitivity and subgroup analyses.

## 2 Materials and methods

### 2.1 Search strategy

We will follow the Preferred Reporting Items for Systematic Reviews and Meta-Analyses (PRISMA) reporting guidelines to conduct this meta-analysis. Two investigators will perform a systematic search of scientific literature in the databases (from conception through June 12, 2021), including PubMed, Embase, and Cochrane Central Register of Controlled Trials. The systematic review protocol has been registered on Open Science Framework registries. This study did not require ethical approval since all analyses were based on previously published studies.

### 2.2 Eligibility criteria and study selection

The intended study inclusion criteria are as following: RCTs of SGLT2 inhibitors in patients with T1DM; studies written in English, and studies with a minimum duration of 12 weeks. Exclusion criteria include case report, review, and nonrandomized controlled trial; studies using SGLT2 inhibitors as monotherapies in the experimental group; and studies in which the placebo group did not receive therapy or placebo. The selection of search literature will be carried out in 2 steps. First, all the titles and abstracts of the search literature are reviewed, and then they are divided into “qualified for full text search” or “not qualified for full text search” categories. Second, all literature classified as “eligible for full document review” is reviewed in detail and then classified as “eligible for meta-analysis” or “ineligible for meta-analysis.”

### 2.3 Data extraction

The records retrieved from the electronic search are imported into the reference management software EndNote X8. Two independent investigators will evaluate all references and extracted data, including study design, sample size and demographics, various treatment strategies, and cardiovascular disease outcomes. The decision to include a study is made by consensus, and any differences between 2 investigators at any stage of the study selection process are arbitrated by a third reviewer and resolved by consensus. Full-text editions of all publications that might qualify for a meta-analysis are scanned and evaluated in detail according to predefined eligibility criteria.

### 2.4 Data analysis

This meta-analysis will be conducted using RevMan statistical software (version 5.3; Nordic Cochrane Center, Copenhagen, Denmark). We will use the Mantel-Haenzel method to calculate the pooled odds ratio. Odds ratio with a 95% confidence interval and mean difference or standardized mean differences with 95% confidence interval will be assessed for dichotomous outcomes or continuous outcomes, respectively. Subgroup analyses will be conducted based on medication for T1DM patients. The heterogeneity will be assessed by using the Q test and *I*^2^ statistic. An *I*^2^ value of <25% is chosen to represent low heterogeneity and an *I*^2^ value of >75% to indicate high heterogeneity. The likelihood of publication bias will be assessed graphically by generating a funnel plot. All reported *P* values are 2-tailed. Variables with *P* values < .05 will be considered statistically significant.

### 2.5 Risk of bias

The risk of bias for each included study will be assessed using the Cochrane Risk of Bias Assessment Tool. The predefined key areas are random sequence generation, allocation concealment, blinding of participants and personnel, blinding of outcome assessment, incomplete outcome data, selective reporting, and other bias. Depending on the articles included, each field is judged to be “low risk of bias” or “unknown risk of bias” or “high risk of bias.”

## 3 Discussion

T1DM accounts for less than 5% of the total diabetes mellitus cases worldwide, affecting approximately 22 million adults and 0.4 million children. SGLT-2 inhibitors have the potential to provide improved glycemic control through a non–pancreatic-dependent mechanism of action.^[5]^ Currently, there are a number of SGLT2 inhibitors that are under development or in clinical trials.^[6–8]^ Prior meta-analyses had established the safety and efficacy of SGLT2 inhibitors in T1DM, but with low level of evidences and inconsistent conclusions.^[9,10]^ However, recently many new RCTs has been published, we hence try to design a study protocol to assess the effect of SGLT2 inhibitors on cardiovascular events via a comprehensive meta-analysis of data from much more RCTs, including sensitivity and subgroup analyses.

## Author contributions

**Conceptualization:** Yanan Huo.

**Data curation:** Yajie Zhang, Ping Gan.

**Formal analysis:** Yajie Zhang, Ping Gan.

**Funding acquisition:** Yanan Huo.

**Investigation:** Yajie Zhang, Ping Gan.

**Methodology:** Yajie Zhang, Ping Gan, Yanan Huo.

**Project administration:** Yanan Huo.

**Software:** Ping Gan, Yajie Zhang.

**Supervision:** Ping Gan, Yanan Huo.

**Validation:** Ping Gan.

**Writing – original draft:** Yajie Zhang.

**Writing – review & editing:** Yanan Huo.
